# *Lactobacillus kefiranofaciens*, the sole dominant and stable bacterial species, exhibits distinct morphotypes upon colonization in Tibetan kefir grains

**DOI:** 10.1016/j.heliyon.2018.e00649

**Published:** 2018-06-06

**Authors:** Xingxing Wang, Jinzhou Xiao, Yusheng Jia, Yingjie Pan, Yongjie Wang

**Affiliations:** a College of Food Science and Technology, Shanghai Ocean University, Shanghai, China; b Laboratory of Quality and Safety Risk Assessment for Aquatic Products on Storage & Preservation (Shanghai), Ministry of Agriculture, China; c Laboratory for Marine Biology and Biotechnology, Qingdao National Laboratory for Marine Science and Technology, Qingdao, China

**Keywords:** Food science, Microbiology

## Abstract

Tibetan kefir grains (TKGs), natural starters for milk fermentation, are believed to comprise diverse microflora of lactic acid and acetic acid bacteria. In order to better understand the bacterial community in TKGs, TKGs that had been cultured continuously either naturally or aseptically for 10 months were subject to analysis using both culture-dependent and various culture-independent methods. Results of DGGE, metagenomics, FISH, qPCR and isolation all demonstrated that *Lactobacillus kefiranofaciens* is the only dominant and stable bacterial species in TKGs regardless of culture conditions and time. FISH and SEM showed that *L. kefiranofaciens* exhibited two distinct morphotypes of short rod (3.0 μm in length) and long rod (10.0 μm in length) upon colonization of either the outer surface or inner component of TKGs, providing evidence for its trophic adaptation to the hollow globular grain structure of TKGs. These findings pave ways for further study of the specific symbiotic interaction between *L. kefiranofaciens* and the dominant *Saccharomyces cerevisiae* yeast in TKGs in vivo.

## 1 Introduction

Tibetan kefir grains (TKGs) originate from Tibet, China. They have been used to ferment cow milk or goat milk into kefir, which is a kind of beneficial beverage. It has been shown that kefir might possess higher nutritional value as well as more health benefits, including enhancing immune system, improving digestive health, and performing antimicrobial, antitumoral and antioxidant activities (Diniz et al., 2003; Rodrigues et al., 2005; Urdaneta et al., 2007; Vinderola et al., 2005), than other milk products.

TKGs contain unique microbial symbionts including both bacteria and yeasts. The microbial community of TKGs constantly undergoes changes, which could mainly be attributed to different sub-culturing conditions. To date, based on various culture-dependent and culture-independent methods, approximately 15 bacterial species have been identified in TKGs collected from different Chinese regions, with most of them belonging to the *Lactobacillus* family (Gao et al., 2012; Huang et al., 2013; Mei et al., 2014; Zheng et al., 2013; Zhou et al., 2009). In addition, other KGs also appear to comprise diverse bacterial species (Nalbantoglu et al., 2014).

In our previous work, the diversity of yeasts in TKGs cultured in our laboratory was investigated (Lu et al., 2014). Interestingly, our results showed that *Saccharomyces cerevisiae* was the predominant yeast species in TKGs, and its total cell number did not exhibit significant changes in response to two different culture conditions (natural and aseptic) and two different sampling time-points (before and after 10 months of continuous cultivation).

In this study, based on culture-dependent and culture-independent methods, e.g., isolation, DGGE, metagenomics, SEM, FISH, qPCR, we confirm that *Lactobacillus kefiranofaciens* is the dominant bacterial species in the same TKGs as analyzed for the diversity of yeasts (Lu et al., 2014) as well as has two distinct morphotypes upon colonization of either the outer surface or inner component of TKGs. These results contribute to better understanding of the unknown symbiotic relationships between yeasts and bacteria in TKGs.

## 2 Materials and methods

### 2.1 TKGs and culture conditions

In the laboratory, TKGs were cultured at 25 °C in pasteurized whole milk (Bright Dairy, Shanghai) that was renewed every 24 h. Two distinct cultivations as described in (Lu et al., 2014) were performed, the naturally cultured TKGs (NCTKGs) and the aseptically cultured TKGs (ACTKGs). The NCTKGs were exposed to potential contamination from environmental microorganisms. In contrast, the ACTKGs were maintained in sterile beakers that were covered with eight layers of sterile gauze, and milk was refreshed in clean bench. Four kinds of samples from the two treatments were collected: NCTKG-1 (N-1) and ACTKG-1 (A-1) representing the samples collected before 10 months of continuous cultivation and NCTKG-2 (N-2) and ACTKG-2 (A-2) collected after 10 months.

### 2.2 DNA extraction of TKGs

TKGs (approximately 14 mm in diameter) growing in renewed milk for 24 h were sampled and washed thoroughly with sterile water to remove any adhered kefir and attached microbes prior to DNA extraction. Approximately 40 mg of biomass from each grain (n = 3) was taken aseptically, pestled with TissueRuptor (Tiangen, Beijing, China) and then suspended in 600 μl of 1.2 M sorbitol buffer. After addition of 50 U lyticase (Tiangen, Beijing, China), the samples were allowed to react for 30 min at 30 °C. The samples were centrifuged at 1500×g for 10 min, and the deposits were resuspended in buffer (20 mM Tris-HCl, 2 mM sodium EDTA, 1.2% Triton×-100) with 20 mg/ml lysozyme (Tiangen, Beijing, China) for 50 min at 37 °C. Subsequently, total DNA was extracted according to the instructions of the Marine Animals DNA Kit (Tiangen, Beijing, China) with a modification involving the addition of 50 μl of (20 mg/ml) Proteinase K solution (Tiangen, Beijing, China).

### 2.3 DGGE analysis of bacterial communities in TKGs

The V3 region of the 16S rRNA gene was amplified using extracted genomic DNA from TKGs (N-1, A-1, N-2, and A-2). PCR was performed in a total reaction volume of 50 μl, containing 25 μl of 2× *Taq* PCR MasterMix (TAKARA, Japan), 1 μl of 10 μM of each primer (V3-F and V3-R) (Leite et al., 2012) (Table 1) and approximately 100 ng template DNA. The PCR program was as follows: 94 °C for 5 min; followed by 30 cycles at 94 °C for 30 s, 55 °C for 30 s, 72 °C for 1 min; and a single final extension step of 72 °C for 10 min. Size and relative amount of PCR products were verified with electrophoresis on 2% (w/v) agarose gel.

**Table 1:** PCR primers used in this study.

Target group	Primer (5′ to 3′)	Product size (bp)	Reference
DGGE			
Bacteria	V3-F-GC^a^: TACGGGAGGCAGCAG	180	Wang et al., 2012
	V3-R: ATTACCGCGGCTGCTGG		
Metagenomic			
Bacteria	V1-F: AGAGTTTGATCCTGGCTCAG	311	Suzuki and Giovannoni, 1996
	V2-R: TGCTGCCTCCCGTAGGAGT		
Plasmid standard			
Bacteria	27-F: AGAGTTTGATCMTGGCTCAG	1,465	Lane, 1991
	1492-R: GGTTACCTTGTTACGACTT		
Real-time qPCR			
Bacteria	338-F: ACTCCTACGGGAGGCAGCAG	181	Einen et al., 2008
	518-R: ATTACCGCGGCTGCTGG		
*L. kefiranofaciens*	LK1-2F: GAGCGGAACCAGCAGAATCA	150	This study
	LK1-2R: GCTGTTCATGCGAACTGCTT		

a GC-clamp at 5′ end: CGCCCGCCGCGCGCGGCGGGCGGGGCGGGGGCACGGGGGGCC.

DGGE was performed under the following conditions: a linear 40–60% denaturant gradient (100% denaturant contained 7 M of urea and 40% of deionized formamide), a constant voltage of 60 V for 16 h (Bio-Rad, USA), and a temperature of 60 °C. The gels were stained in 1× SYBR I in TAE buffer (Invitrogen, USA) for 20 min, and then visualized by using Bio-Rad Gel Doc XR+ imaging system.

### 2.4 Metagenomic analysis of bacterial communities in TKGs

The TKGs (N-1, N-2, A-1 and A-2) 16S rRNA gene sequence data of the V1-V2 region (Suzuki and Giovannoni, 1996) (Table 1) was generated with an Illumina MiSeq platform and was processed using the Mothur software package (v.1.35.1) (Kozich et al., 2013). Briefly, 250-bp read pairs were assembled into contigs based on read alignment. Any contigs that exhibited homopolymer of more than 8 nucleotides, contained any ambiguous bases, failed to align to V1-V2 region or were flagged as possible chimeras by UCHIME (Edgar et al., 2011) were removed prior to further analysis. Contigs were then aligned to a SILVA reference (Pruesse et al., 2007) and pre-clustered by combining the numbers of sequences that differed by 2 or fewer nucleotides.

Sequence classification was performed using a naive Bayesian classifier trained against Ribosomal Database Project (RDP) training set (version 9) (Cole et al., 2014) with 80% of bootstrap confidence threshold. Sequences that were unassigned (including unidentifiable bacteria) or classified as *Archaea*, *Eukaryota*, chloroplasts, or mitochondria were discarded. Sequences affiliated with *Lactobacillus* were compared with NCBI bacterial 16S ribosomal sequence database using BLASTn (E-value < 10^−5^) to identify the specific species.

Finally, sequences were clustered into operational taxonomic units (OTUs) based on pairwise identity of 97% and abundance of more than 0.005%. Similarities among samples were evaluated with collector's curve of OTUs, Chao1 richness and inverse Simpson index.

### 2.5 Isolation and identification of *Lactobacillus kefiranofaciens* in TKGs

TKGs (A-2) were sampled and washed with sterile water to remove clotted milk from the grain surfaces. Approximately, 0.1 g grains were grinded aseptically with TissueRuptor (Tiangen, Beijing, China) and then suspended in 1 ml of 0.9% saline. One hundred microliter of aliquot of the suspension was spread onto six MRS agar plates (pH 5.0 and 1.5 g of agar per 100 ml), which were then incubated at 30 °C for 7 days under an anaerobic condition (Anaero Pack-Anaero kit, Mitsubishi Gas Chemical CO Inc., Tokyo, Japan).

Eighteen colonies (three per plate) were randomly selected and then subjected to colony PCR assay. The primers used were 27-F and 1492-R (Lane, 1991) as showed in Table 1. PCR was performed in a total reaction volume of 25 μl, containing 12.5 μl of 2× *Taq* PCR MasterMix (TAKARA, Japan), 1 μl of 10 μM each primer (Table 1) and the bacterial cells as template. The PCR program was as follows: 95 °C for 5 min; followed by 30 cycles at 94 °C for 30 s, 55 °C for 30 s, 72 °C for 1 min; and a final extension step at 72 °C for 10 min. The PCR products were purified and sequenced (Sangon, Shanghai, China), and the sequences were analyzed with BLASTn (http://blast.ncbi.nlm.nih.gov/Blast.cgi).

Isolation of the genomic DNA from *L. kefiranofaciens* cells was performed according to the instructions described in the Bacterial Genomic DNA Extraction kit (Tiangen, Beijing). The V3 region of the 16S rRNA gene was amplified for DGGE analysis using the same method described above.

### 2.6 SYBR Green I staining of *L. kefiranofaciens*

*L. kefiranofaciens* strains were cultured in MRS broth (pH 5.0) for 5 or 7 days under anaerobic condition (Anaero Pack-Anaero kit, Japan). One milliliter of *L. kefiranofaciens* cells was centrifuged at 6000× g for 1 min. The cell pellets were re-suspended in PBS solution and was fixed in 4% of formaldehyde for 14 h. The fixed *L. kefiranofaciens* cells were filtered through a 0.2 μm GTTP membrane (Merck Millipore), stained with 1 × SYBR Green I (Solarbio, Beijing, China) for 15 min, and observed under an epifluorescence microscope (Zeiss Axiophot, Germany).

### 2.7 Scanning electron microscope (SEM) observation

Procedures for preparing *L. kefiranofaciens* cells were the same as described above, except that the cells were fixed in 2.5% of glutaraldehyde solution for 2 h at 4 °C. The cells were then washed in 0.1 M of phosphate buffer for 3 times, each 15 min, and post-fixed in 1% of osmium tetroxide for 1 h at 4 °C, followed by 3 washes with 0.1 of M phosphate buffer and then with a series of ethanol (50%, 70%, 80%, 90%, 95%, 100% and 100% (v/v) containing anhydrous sodium sulfate) for 15 min at room temperature.

The fixed *L. kefiranofaciens* cells were desiccated in a mix solution (isoamyl acetate: ethanol = 1:1) and in isoamyl acetate for 30 min, respectively, then dried in a critical-point dryer (HCP-2 HITACHI, Japan) for 5 h. The dehydrated samples were displayed on specimen stubs with a double-sided adhesive tape. The samples were observed using a SEM (S-4800 HITACHI, Japan) after being coated with gold using a sputter coater (Cressingtom 108 auto).

Methods for sample preparation and SEM observation of TKGs were the same as described in our previous work (Lu et al., 2014).

### 2.8 Fluorescence in situ hybridization (FISH)

Probes used in this study are shown in Table 2. The probe EUB338 targets most bacterial species (Amann and Fuchs, 2008). As for *L. kefiranofaciens*, specific probe was designed based on the 16S rRNA gene sequence obtained in this study and its specificity was verified using BLASTn program (http://blast.ncbi.nlm.nih.gov).

**Table 2:** Fluorescent probes used in this study.

Probe	Target	Sequence (5′-3′)	Fluorescein	Formamide (%)
EUB338	Bacteria	GCTGCCTCCCGTAGGAGT	5′Cy3	25
Lkb	*L. kefiranofaciens*	CCACCGCTACACATGGAGTTCTAC	5′FITH	25

Preparation of frozen thin sections of TKGs for FISH was performed according to the methods described previously (Lu et al., 2014). After treated with lysozyme solution (10 g/l in Tris-HCl pH 6.5) at 37 °C for 10 min, TKG thin sections were hybridized with probes EUB338 and Lbk, which are labeled with different fluorophores, at 46 °C for 3 h and then washed at 48 °C for 30 min. Finally, the samples were stained with Vectashield-DAPI (Vector Laboratories, Burlingame, USA) and observed under a Zeiss Axiophot epifluorescence microscope with filter sets for DAPI, Cy3, and FITC.

### 2.9 Real-time quantitative PCR

Primers for real-time qPCR are shown in Table 1. The specific primers for *L. kefiranofaciens* were designed based on its full length 16S rRNA gene sequence using Geneious 6.1.8 software (Biomatters). Specificity of primer set was confirmed with the GenBank database using the Primer-BLAST program (http://www.ncbi.nlm.nih.gov/tools/primer-blast).

Plasmid standards used in this study were prepared as described in (Lu et al., 2014). Briefly, the total genomic DNA of *L. kefiranofaciens* was extracted according to the methods described above. The primer set of 27F/1492R (Lane, 1991) was used to amplify the full length 16S rRNA gene of *L. kefiranofaciens*. PCR was performed in a total reaction volume of 50 μl containing 25 μl of 2× *Taq* PCR MasterMix (Tiangen, Beijing, China), 1 μl of 10 μM of each primer, and approximately 100 ng of DNA template. The PCR program was as follows: 94 °C for 5 min, followed by 35 cycles at 94 °C for 30 s, 55 °C for 30 s, 72 °C for 1 min, and a final extension step of 72 °C for 10 min. The amplified PCR products were purified using the PCR Fragment Purification Kit (Tiangen, Beijing, China) and then cloned with the pGM-T Cloning Kit (Tiangen, Beijing, China). The recombinant plasmids were transformed into competent *Escherichia coli* T0P 10 cells following supplier's instructions. Positive clones were identified by colony PCR, 2.0% agarose gel electrophoresis and sequencing. Sequence similarity of obtained sequences was analyzed by BLASTn (http://blast.ncbi.nlm.nih.gov/Blast.cgi). The positive clones carrying the 16S rRNA gene fragments of *L. kefiranofaciens* were cultivated. The recombinant plasmids were extracted using the QIAprep Spin Miniprep Kit (Qiagen, Hilden, Germany) and quantified by BioTek Synergy 2.0 (BioTek, USA) and Quant-iT PicoGreen dsDNA Reagent and Kit (Invitrogen, USA).

The total genomic DNA of kefir grains was extracted and quantified according to the methods described above. Then, a serial dilution was carried out to achieve a final concentration ranging from 0.1 to 1.0 ng/μl. The reaction mix (20 μl) contained 2.0 μl of template (plasmid standard or kefir grain genomic DNA), 0.4 μl of each of the specific primers, 10 μl of SYBR Premix Ex *Taq* II (2×) (Takara, China), and 0.4 μl of Rox Reference Dye (50×). The following qPCR thermal program was used: 95 °C for 10 min, followed by 40 cycles at 94 °C for 15 s, 59 °C for 30 s, and 72 °C for 30 s. The amplification reaction was performed in an ABI 7500 Fast real-time PCR system along with version 2.0.1 of the software (Applied Biosystems, USA). Quantification of total bacteria (Einen et al., 2008) and *L. kefiranofaciens* (Table 1) were performed in three parallel experiments. The level of statistical significance was set at *p* value of <0.01.

### 2.10 Nucleotide sequence accession numbers

High-throughput sequencing reads were deposited into the SRA database under their corresponding accession numbers (SRR5039623–SRR5039626).

## 3 Results

### 3.1 DGGE analysis of the bacterial community in TKGs

**Fig. 1:**
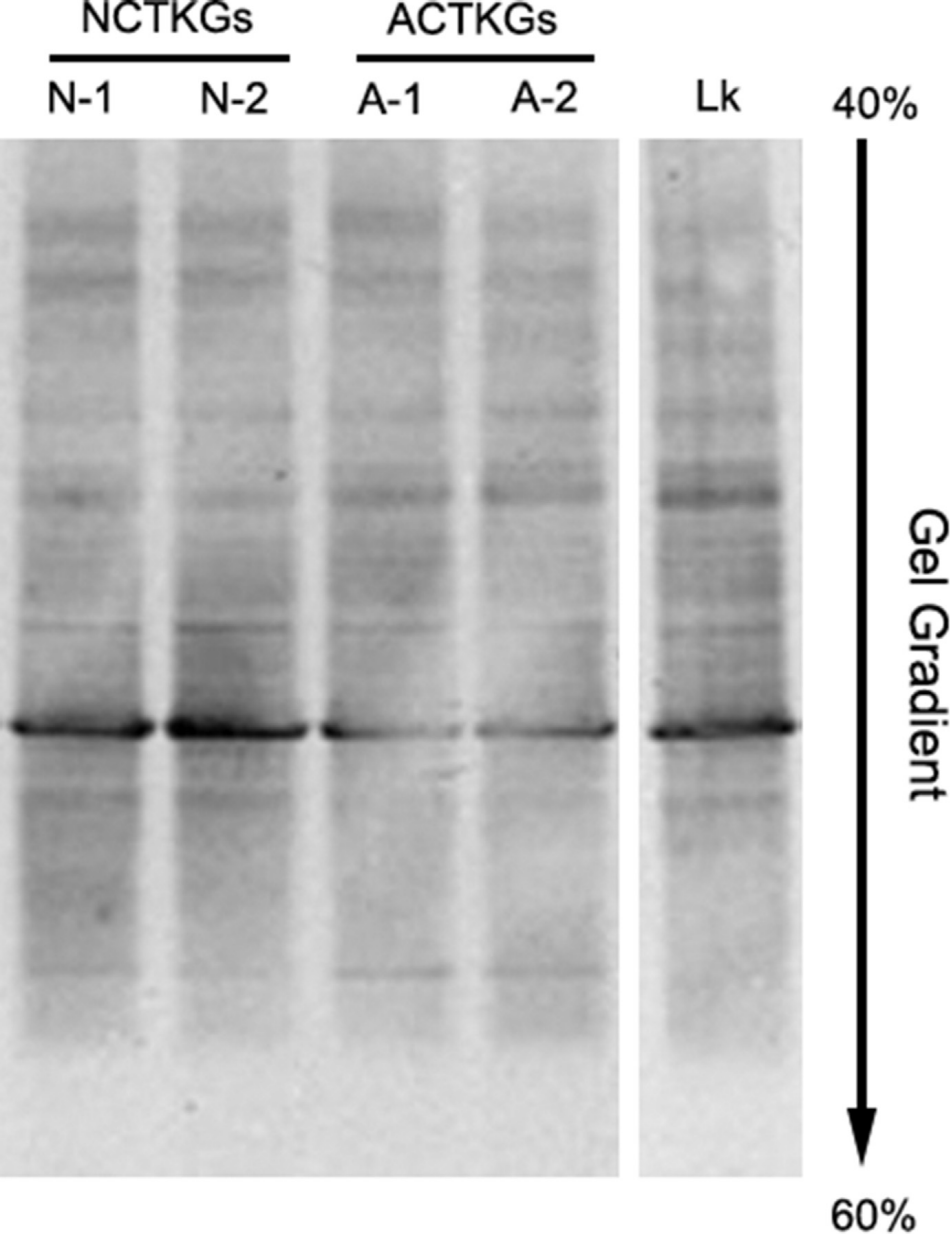
DGGE fingerprints of bacterial community in Tibetan kefir grains. N-1: NCTKG-1, N-2: NCTKG-2, A-1: ACTKG-1, A-2: ACTKG-2. A-2 and N-2 were sampled after 10 more months of continuous cultivation in comparison to N-1 and A-1. Lk: Pure culture of *L. kefiranofaciens.* These abbreviations are applicable to all subsequent figure legends. Three parallels for each sample were analyzed and one of them is shown here.

DGGE was performed in order to identify the bacterial community in TKGs. As shown in Fig. 1, a single, strong and sharp band that appeared at the same position on the gel was observed in each of the four samples of N-1, N-2, A-1 and A-2 (Three parallels for each sample were analyzed). It suggests that the dominant bacterial species in TKGs were stable under two culture conditions (nature and sterile) throughout a time span of 10 months.

### 3.2 Metagenomic analysis of bacterial community in TKGs

To get a thorough understanding of the bacterial diversity in TKGs, samples of N-1, N-2, A-1 and A-2 were further analyzed with 16S metagenomics. Given that no differences in DGGE profiles were observed for three parallel samples, only one of them was subjected to sequencing analysis. The number of obtained sequences and the sequencing coverage for each sample were presented in Table 3.

**Table 3:** Sequence data, coverage, OTU richness and diversity index of the 16S rRNA gene metagenomic sequencing.

Label	Sample	Raw data	Clean data	Coverage	OTUs	Invsimpson	Invsimpson_lci	Invsimpson_hci
0.03	N-1	21,592	13,222	0.998389	65	1.065626	1.059133	1.072198
0.03	N-2	24,693	15,648	0.998772	60	1.123459	1.114302	1.132769
0.03	A-1	27,770	18,597	0.998005	61	1.067778	1.061175	1.074464
0.03	A-2	25,043	15,870	0.998772	59	1.053267	1.047471	1.059127

**Fig. 2:**
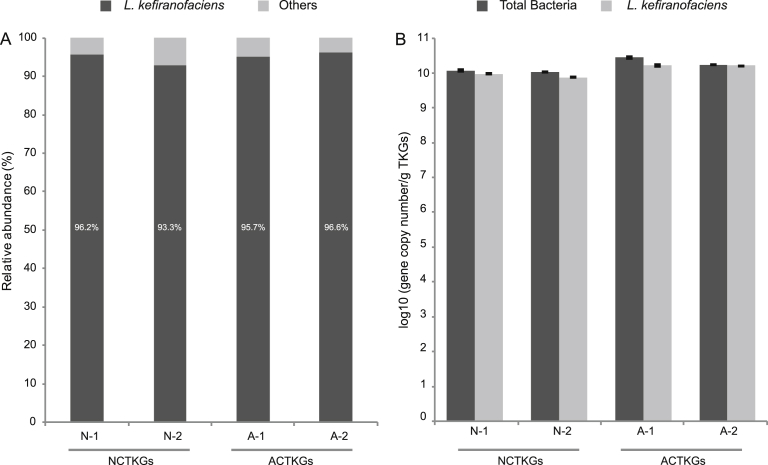
The relative abundance and stabilization of *L. kefiranofaciens* in TKGs. The 16S rRNA gene metagenomic analysis (A) and the Real-time qPCR assay (B). Standard deviations are shown as error bars.

According to Bayesian classifier, the majority of the sequences from the four samples were assigned to the genus of *Lactobacillus* (≈97%). BlastN similarity search showed that *L. kefiranofaciens* was the only predominant species assigned to the *Lactobacillus*-associated sequences (Fig. 2A), indicating that it was the dominant bacterial species in TKGs. In addition, there was no significant difference in the relative abundance of this species among the four samples, suggesting that it remained stable in TKGs under different culture conditions and time.

**Fig. 3:**
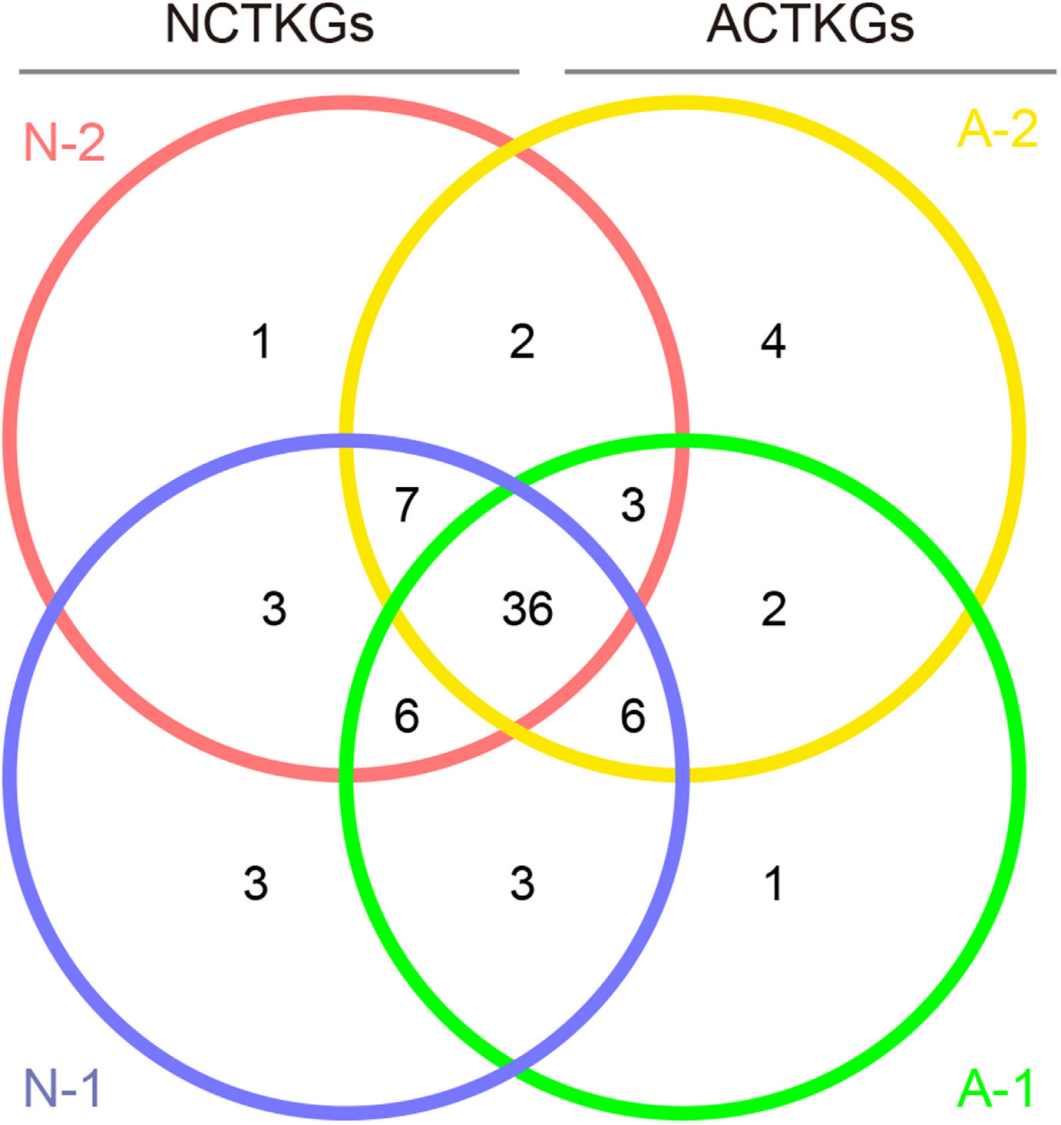
Venn diagram of the bacterial 16S rRNA gene metagenomic datasets from Tibetan kefir grains at 97% similarity. The majority of the OTUs were common to all four samples.

To mitigate the effects of potential uneven sampling, all samples were reduced to 13,032 sequences prior to analysis of OTU richness and species diversity. A total of 80 OTUs were assigned to the four samples at 97% sequence similarity, and 36 OTUs were common among the samples (Fig. 3). The results suggest that both the abundance and diversity of bacterial species were similar among these four samples.

**Fig. 4:**
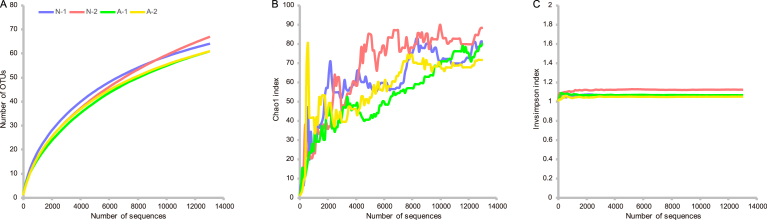
Bacterial community similarity in different samples of Tibetan kefir grains based on the 16S rRNA gene surveys. Rarefaction curve (A), Chao1 (B) and invsimpson index (C).

Both the Chao1 and OTU rarefaction curves appeared to be approaching parallel to the horizontal axis in all samples (Fig. 4), indicating a sufficient sample size for adequate assessment of species richness. The substantial coincidence between Chao1 and invsimpson index curves suggests the similarity of species abundance across the four samples. In addition, the coincidence between invsimpson and the rarefaction curves showed that species evenness is almost identical among the four samples.

Similarity in the OTU structures of the samples was determined using jclass calculator and the statistical significance of clustering was evaluated by various algorithms. Our results revealed that the difference between two culture treatments (N-1 and N-2 vs. A-1 and A-2) as well as that between the time span of 10 months (N-1 and A-1 vs. N-2 and A-2) were not statistically significant (Table 4), indicating the absence of variance across the samples.

**Table 4:** Parsimony, weighted and unweighted unifrac analysis of the bacterial species similarity in kefir grains under different culture condition and time.

Groups	ParsScore	ParsSig	WScore	UWScore
Before vs. after 10 months	1	0.32	0.519911	0.534577
Natural vs. aseptic condition	2	1	0.511601	0.526521

Taken together, we conclude that culture condition and time span had little impact on the bacterial diversity in TKGs. This finding is in concordance with the DGGE results presented above.

### 3.3 Isolation and identification of *L. kefiranofaciens* in TKGs

Isolation and cultivation of *L. kefiranofaciens* was performed to verify its presence in TKGs. Since the bacterial community in the four samples of TKGs were almost identical based on the results of DGGE and 16S metagenomics analyses, only the samples of A-2 were subjected to bacterial isolation. No morphological differences were observed among the obtained colonies on MRS agar plates (n = 6). The colony PCR products were sequenced and analyzed with BLASTN. All 18 sequences shared 99–100% of similarity with the 16S rRNA gene of *L. kefiranofaciens.* In addition, the DGGE profiles of the *L. kefiranofaciens* cells were almost identical to that of TKGs (Fig. 1). Therefore, it is conceivable to conclude that *L. kefiranofaciens* is a stable dominant bacterial species in TKGs.

**Fig. 5:**
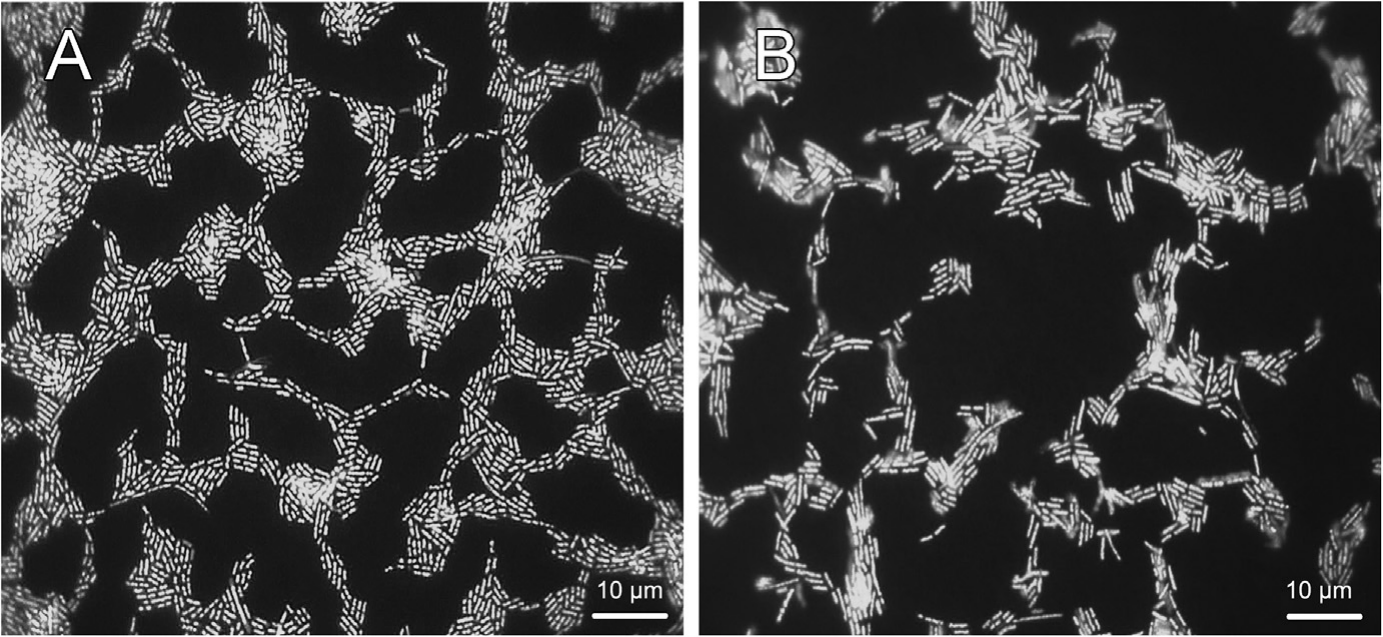
SYBR Green I staining of *L. kefiranofaciens* cells. *L. kefiranofaciens* cells were cultured for 5 (A) and 7 (B) days.

In addition, *L. kefiranofaciens* cells appeared as rod-shaped cells after SYBR Green I staining, and they were slightly longer in shape after 7 days of culture in MRS broth compared with those from a 5-day culture (Fig. 5).

### 3.4 SEM observation of *L. kefiranofaciens* and TKGs

**Fig. 6:**
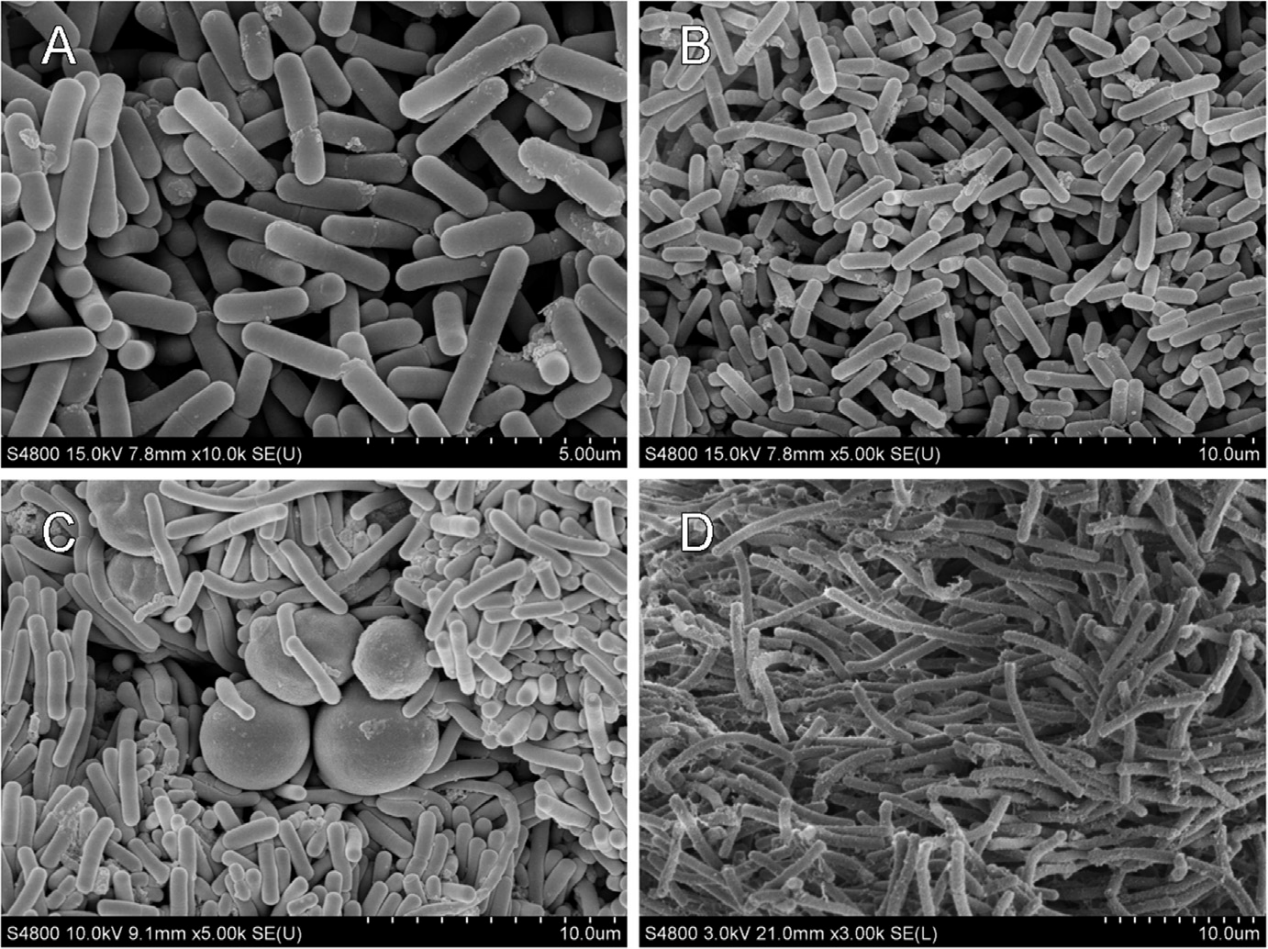
Scanning electron micrographs of *L. kefiranofaciens* cells and TKGs. *L. kefiranofaciens* (A, B); the outer surface (C) and the inner cross-section (D) of the TKGs.

Under SEM, the pure culture cells of *L. kefiranofaciens* are rod-shaped (1.7–2.5 × 0.5–0.6 μm) (Fig. 6A and B). In contrast, two different size of rod-shaped cells were observed in TKGs, approximately 3.0 μm in length on the outside surface (Fig. 6C) and 10.0 μm in length in the inner parts (Fig. 6D).

### 3.5 FISH analysis of *L. kefiranofaciens* in TKGs

**Fig. 7:**
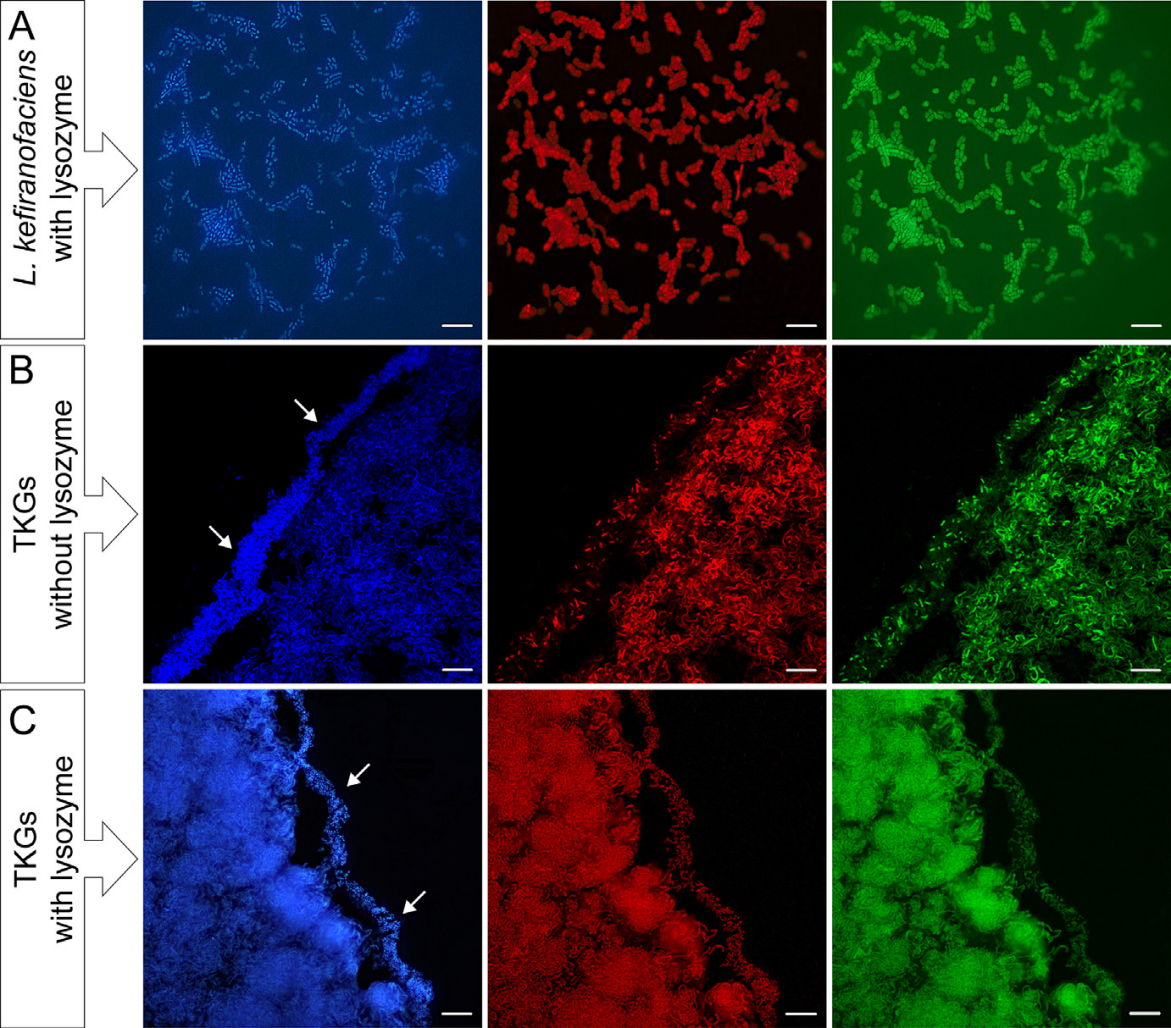
FISH analysis of *L. kefiranofaciens* cells and TKGs. Blue: DAPI-staining; red: hybridization with the bacteria specific probe EUB338; green: hybridization with the *L. kefiranofaciens* specific probe Lbk. Arrow: the short rod shaped *L. kefiranofaciens* on the surface layer of TKGs. Scale bar = 10 μm.

Probe specificity was verified *in silico* as well as through hybridization with pure culture of *L. kefiranofaciens.* Both BLASTN similarity search and SILVA database check confirmed that the probe Lbk is 100% specific for *L. kefiranofaciens* only. However, whole cell hybridization initially failed for both probes, EUB338 and Lbk. Nevertheless, after treatment of the bacterial cells with lysozyme, all DAPI-stained cells showed positive signals with both bacteria-specific and *L. kefiranofaciens*-specific probes (Fig. 7A). It indicates that the cell walls of *L. kefiranofaciens* are too thick to be penetrated by probes.

To avoid potential unspecific hybridization, a series of experiments were performed with the strains of *L. kefiranofaciens* at various concentrations of formamide, from 10 to 70%, in hybridization buffer and washing buffer. Finally, the high stringency at 25% of formamide was determined to be the ideal hybridization condition and was employed in subsequent FISH analysis of thin sections of the four samples of TKGs. All the bacterial cells, including the short rods on the outer surface and the long rods in the inner parts of TKGs, were positive for both the EUB338 and Lbk probes (Fig. 7C). It confirmed that the bacteria in TKGs were, in fact, *L. kefiranofaciens*.

### 3.6 qPCR analysis of *L. kefiranofaciens* in TKGs

Furthermore, no significant variation in copy number per gram of TKGs was observed between total bacteria and *L. kefiranofaciens* in both the NCTKGs and the ACTKGs before and after 10 months of continuous cultivation (Fig. 2B): (1.12 ± 0.06) × 10^10^ (total bacteria) vs. (0.91 ± 0.29) × 10^10^ (N-1) and (1.05 ± 0.04) × 10^10^ (total bacteria) vs. (0.75 ± 0.31) × 10^10^ (N-2); (2.75 ± 0.12) × 10^10^ (total bacteria) vs. (1.62 ± 0.08) × 10^10^ (A-1) and (1.72 ± 0.02) × 10^10^ (total bacteria) vs. (1.63 ± 0.04) × 10^10^ (A-2). These results further supported that *L. kefiranofaciens* is the only dominant bacterial species in TKGs and it remains stable under different culture conditions and time spans.

## 4 Discussion

In this study, based on both culture-dependent and various culture-independent methods of DGGE, metagenomics, qPCR and FISH, we found that *L. kefiranofaciens* is the only dominant bacterial species in TKGs cultured in different conditions and time span. *L. kefiranofaciens* is also often identified as one of the dominant bacterial species present in KGs of different geographic locations and sub-culture conditions (Chen and Chen, 2013; Chen et al., 2013; Nalbantoglu et al., 2014; Wang et al., 2012; Zhou et al., 2009). However, in contrast to previous findings that showed a diverse dominant bacterial community in grains (Gao et al., 2013; Garofalo et al., 2015; Kesmen and Kacmaz, 2011; Leite et al., 2012; Marsh et al., 2013; Nalbantoglu et al., 2014), our results indicate that *L. kefiranofaciens*, possibly serving as the constitutive resident, is more resistant to variations in culture conditions and plays a more important role in the formation and stability of TKGs in comparison to other bacterial species.

Unexpectedly, *L. kefiranofaciens* displayed two distinct morphological characteristics of short and long rods depending on its location within TKGs. This unique phenomenon has not been reported before and could be attributed to the restricted amount of available nutrients that are accessible to cells trapped in the inner grains, which could consequently lead to discrepancy in phases of cell growth in different parts of the grains, for example, exponential phase on the outside surface and stationary and/or death phases within the grains. Notably, longer or larger cell shape is often one of the typical features of bacterial cells that are approaching stationary and death phases (Madigan et al., 2010). Coincidentally, we observed that *L. kefiranofaciens* cells cultured in MRS liquid media for 7 days were distinctively longer than those cultured for 5 days, although the cells located on the grain surface were similar in size (Figs. 5 and 6). In addition, without lysozyme treatment, a vast majority of cells from 5 day culture hybridized with neither the bacteria-specific nor the *L. kefiranofaciens*-specific probe, while some of the 7 day cells showed positive FISH signals after hybridization (data not shown). Furthermore, the short *L. kefiranofaciens* cells on grain surface revealed almost no signals after hybridization with these two probes, however, most of the long cells in the inner parts of grains demonstrated positive hybridization (Fig. 7B). In contrast, after lysozyme digestion, both the short and long *L. kefiranofaciens* cells in the pure culture as well as in TKGs were all positive upon hybridization with the probes (Fig. 7A and C). These results suggest that the cell walls of the long *L. kefiranofaciens* cells within the grains are more permeable than that of the short *L. kefiranofaciens* cells on grain surface, which is likely a phenomenon resulted from the physical and structural adaption of the *L. kefiranofaciens* to the limitation of available nutrients. Our results also suggest that growth of the grains likely starts from the outside of the grains inward.

In 2013, Mendes and coauthors reported that *L. delbrueckii* subsp. *bulgaricus* cells grown in co-culture with *S. cerevisiae* yeast were significantly shorter than those grown in pure culture (Mendes et al., 2013), and that metabolism of yeast is supposedly in charge of the morphological changes of *L. delbrueckii* cells in the co-culture. However, in our study, both the short and long rod-shaped *L. kefiranofaciens* cells were observed to co-exist with yeasts in TKGs (Lu et al., 2014). Therefore, the variations in *L. kefiranofaciens* cell size are unlikely to be directly affected by the growth of yeast in grains.

Additionally, in our previous work, three genera of yeast species (*S. cerevisiae*, *Kluyveromyces marxianus*, *K. lactis* and *Yarrowia lipolytica*) were found in TKGs cultured either naturally or aseptically (Lu et al., 2014), and *S. cerevisiae* yeast is the dominant stable yeast species in TKGs throughout 10 months of continuous cultivation. Interestingly, similar to *S. cerevisiae*, *L. kefiranofaciens* is confirmed to be the dominant stable bacterial species in TKGs in this study. Notably, *L. kefiranofaciens* as one of the predominant bacterial species was observed in other milk KGs, along with different yeast partners, e.g., *Dekkera anomala* or *Naumovozyma* spp. and *Kazachastania khefir*, instead of *S. cerevisiae* (Ferreira et al., 2010; Garofalo et al., 2015). It suggests that *L. kefiranofaciens* seems to be quite versatile in terms of compatibility with different yeast partners.

In conclusion, *L. kefiranofaciens*, the only dominant bacterial species in TKGs, demonstrates a steady-state relationship with the dominant yeast species of *S. cerevisiae* in Tibetan kefir grains. The potential symbiotic interaction between yeasts and *Lactobacilli* in other milk kefir grains as well as the effects of their metabolism and nutrient intake could be applied to TKGs as well (Chao and Reilly, 1972; Megee et al., 1972; Nijkamp et al., 2012; Shindala et al., 1965; van de Guchte et al., 2006). Future investigations are necessary in order to better understand the associations between *L. kefiranofaciens* and *S. cerevisiae*, which will shed lights on the formation and growth of grains.

## Declarations

### Author contribution statement

Xingxing Wang: Performed the experiments; Wrote the paper.

Jinzhou Xiao: Analyzed and interpreted the data.

Yusheng Jia: Performed the experiments.

Yingjie Pan: Contributed reagents, materials, analysis tools or data.

Yongjie Wang: Conceived and designed the experiments; Analyzed and interpreted the data; Wrote the paper.

### Funding statement

This work was partially supported by the National Natural Science Foundation of China (41376135, 31570112).

### Competing interest statement

The authors declare no conflict of interest.

### Additional information

Data associated with this study (high-throughput sequencing reads) has been deposited at into the SRA database under the accession numbers SRR5039623–SRR5039626.
